# Genomic Survey, Characterization, and Expression Profile Analysis of the SBP Genes in Pineapple (*Ananas comosus* L.)

**DOI:** 10.1155/2017/1032846

**Published:** 2017-09-29

**Authors:** Hina Ali, Yanhui Liu, Syed Muhammad Azam, Zia ur Rahman, S. V. G. N. Priyadarshani, Weimin Li, Xinyu Huang, Bingyan Hu, Junjie Xiong, Umair Ali, Yuan Qin

**Affiliations:** ^1^Fujian Provincial Key Laboratory of Haixia Applied Plant Systems Biology; Key Lab of Genetics, Breeding and Multiple Utilization of Crops, Ministry of Education; State Key Laboratory of Ecological Pest Control for Fujian and Taiwan Crops; Center for Genomics and Biotechnology; College of Life Science, Fujian Agriculture and Forestry University, Fuzhou, Fujian Province 350002, China; ^2^College of Crop Sciences, Fujian Agriculture and Forestry University, Fuzhou, Fujian 350002, China; ^3^College of Resources and Environment, Fujian Agriculture and Forestry University, Fuzhou, Fujian 350002, China; ^4^Abasyn University, Peshawar, 25000 KPK, Pakistan

## Abstract

Gene expression is regulated by transcription factors, which play many significant developmental processes. SQUAMOSA promoter-binding proteins (SBP) perform a variety of regulatory functions in leaf, flower, and fruit development, plant architecture, and sporogenesis. 16 *SBP* genes were identified in pineapple and were divided into four groups on basis of phylogenetic analysis. Five paralogs in pineapple for *SBP* genes were identified with Ka/Ks ratio varied from 0.20 for *AcSBP14* and *AcSBP15* to 0.36 for *AcSBP6* and *AcSBP16*, respectively. 16 *SBP* genes were located on 12 chromosomes out of 25 pineapple chromosomes with highly conserved protein sequence structures. The isoionic points of SBP ranged from 6.05 to 9.57, while molecular weight varied from 22.7 to 121.9 kD. Expression profiles of *SBP* genes revealed that *AcSBP7* and *AcSBP15* (leaf), *AcSBP13*, *AcSBP12*, *AcSBP8*, *AcSBP16*, *AcSBP9*, and *AcSBP11* (sepal), *AcSBP6*, *AcSBP4*, and *AcSBP10* (stamen), *AcSBP14*, *AcSBP1*, and *AcSBP5* (fruit) while the rest of genes showed low expression in studied tissues. Four genes, that is, *AcSBP11*, *AcSBP6*, *AcSBP4*, and *AcSBP12*, were highly expressed at 4°C, while *AcSBP16* were upregulated at 45°C. RNA-Seq was validated through qRT-PCR for some genes. Salt stress-induced expression of two genes, that is, *AcSBP7* and *AcSBP14*, while in drought stress, *AcSBP12* and *AcSBP15* were highly expressed. Our study lays a foundation for further gene function and expression studies of *SBP* genes in pineapple.

## 1. Introduction

Pineapple (*Ananas comosus*) is a tropical plant with edible multiple fruits, can be consumed fresh, cooked, juiced, or preserved and is the most economically significant plant in the Bromeliaceae family [1, 2]. Pineapple fruits are important source of vitamins such as vitamin A and vitamin B1. In addition, it also contains a protein-digesting enzyme bromelain, a mixture of proteolytic enzymes with potential that may be used as a therapeutic agent for a variety of clinical disorders in the future [3].

Gene expression is regulated by transcription factors (TFs). Transcription factors are proteins of sequence-specific DNA binding with capability of activating and/or repressing transcription (Luscombe et al. 2000). In plants including the pineapple, transcription factors play essential roles in the regulation networks of many significant developmental processes. Genes in the SQUAMOSA promoter-binding protein (SBP-box) gene family encode SBP proteins, which perform a variety of regulatory functions that are involved in leaf development, vegetative phase change, flower and fruit development, plant architecture, sporogenesis, gibberellic acid signaling, and toxin response [4]. The SBP domain is a highly conserved DNA-binding domain and contains 76 amino acids and two zinc-binding sites, Cys-Cys-His-Cys and Cys-Cys-Cys-His [5, 6]. *AmSBP1* and *AmSBP2* were identified as the first SBP-box genes in *Antirrhinum majus* discovered on the basis of their ability to bind with the promoter sequence of the floral meristem [5]. The first identified SBP-box gene in *Arabidopsis* was SPL3 (SQUAMOSA promoter-binding protein-like 3), which has an essential role in the floral transition [7].

Later, SBP-box genes were identified, isolated, and characterized in many plants, including Arabidopsis [6], silver birch [8], *Salvia miltiorrhiza* [9], rice [10], maize [11], tomato [12], grape [13], and *Gossypium hirsutum* [14]. Furthermore, recent studies showed that SBP-box genes show response to biotic and abiotic stresses and are also involved in signaling pathways in many species, for instance, *AtSPL14* displaying sensitivity to the programmed cell death, inducing fungal toxin fumonisin B1 [15]. In grapes, *VpSBP5* has the ability to show resistance to *Erysiphenecator* by activating the SA-induced systemic acquired resistance pathway and MeJA-induced wound signaling pathway [13]. Recently, it is considered that SBP-box genes are involved in the defense mechanism of *Phytophthora* blight in pepper.

The growth and development of pineapple can be affected by stress conditions such as soil type, temperature, salinity, drought, cold, and water. Importantly, gene expression is essential also for organism growth and development of the plant which could be deregulated under the stress conditions. Abiotic stresses adversely affect the growth and productivity of plants. Generally, plants are exposed to abiotic stresses like drought, salinity, low and high temperature, salt, and chemical stresses. Genetic stability and productivity of crops are affected by all these stresses. More than 50% of crop yield is reduced by abiotic stresses worldwide [16]. On the basis of genome sequence analysis, we identified 16 SBP genes in pineapple and divided it into seven groups to show the phylogenetic relationship with rice and *Arabidopsis*. Also, this study helps to identify the gene structure of SBP gene family, phylogenetic study, protein motifs, gene location on chromosomes, and RNA-Seq analysis for different tissues. Under four abiotic stresses (cold, drought, salt, and heat), the gene expression profiles were assessed. This study will help to identify SBP genes which are showing responses to different stress conditions and can be utilized to create abiotic stress resistant varieties through breeding programs to address the issue of yield loss due to abiotic stress. The effect of SBP proteins on gene expression is well characterized in other plants under stress conditions but is yet to be characterized in pineapple. Therefore, the present study was conducted to characterize SBP genes and the impact of SPB proteins on the gene expression in pineapple under abiotic stress conditions.

## 2. Material and Methods

### 2.1. Genome-Wide Identification of SQUAMOSA (SBP) Gene Family Homologue Sequences

To identify the SBP gene family in pineapple (*Ananas comosus*), the Hidden Markov Model (HMM) profile of the SBP domain (PF03110) was downloaded from the Pfam database. The SBP protein sequences of pineapple, *Arabidopsis thaliana*, and rice were obtained from the Phytozome, UniPort, and RGAP databases, respectively [17]. For the identification of SBP transcription factor-coding genes of *Ananas comosus*, the Hidden Markov Model (HMM) profile of the SBP domain was used to query pineapple (*Ananas comosus*) genome database as well as nonredundant protein database with the help of the BLAST program. Primers were designed to amplify the sequences from PrimerQuest tool software.

### 2.2. Characteristics of SBP Genes in Pineapple

To identify the characteristics of SBP genes in pineapple, ExPASy server was used to calculate the isoionic point (IP) and molecular weights of SBP genes, while the number of amino acids, introns, exons, and open reading frame (ORF) was calculated manually.

### 2.3. Phylogenetic Analysis

Multiple sequence alignments were carried out based on amino acid sequences of SBP gene family using MUSCLE software. To study the evolutionary relationship of SBP gene family in pineapple with *Arabidopsis thaliana* and rice, a phylogenetic tree was constructed based on sequence alignments using RAxML and the maximum likelihood method with the following parameters: JTT model, pair wise gap deletion, and 1000 bootstraps [18]. Furthermore, for tree construction maximum likelihood, minimal evolution and PhyML methods were also applied to validate the results of the neighbour-joining (NJ) method. To find out phylogenetic relationship, Ka/Ks values of SBP genes were also conducted.

### 2.4. Calculation of Ka/Ks Values

Pairwise alignment of SBP gene encoding sequences of the orthologous and paralogous pairs was carried out using bio-pipeline-master software to calculate Ka/Ks value (https://github.com/tanghaibao/bio-pipeline/tree/master/synonymous_calculation).

### 2.5. SBP Gene Location on Chromosomes

To derive the location of SBP genes on chromosomes, genes were mapped on chromosomes using *Mapinspect* software according to the start site (first nucleotide) of gene in pineapple. Start points and stop points of SBP genes were calculated from pineapple genome.

### 2.6. Gene Structure and Conserved Motif Analysis

The structural organizations of exon-intron of SBP gene family in pineapple were determined using the Gene Structure Display Server. Furthermore, by computer alignments and manual analysis, the positions of exons, SBP domains, and CDS were identified [19]. Subsequently, MEME program was employed to identify the motifs of SBP protein sequences in pineapple genome, with the motif length set to 6–100 and motif sites to 2–120. The maximum number of motifs was set to 10. The distribution of one single motif was “any number of repetitions,” and the other parameter was “search-given strand only.”

### 2.7. Plant Material and Growth Conditions

In this study, pineapple (*Ananas comosus)* variety MD2 was used, which are provided by the Haixia Institute of Science and Technology, Center for Genomics and Biotechnology, Fujian Agriculture and Forestry University, Fujian, China. Pineapple plants were grown in medium plastic pots containing soil and were placed in a greenhouse at 30°C with light availability of 60–70 mMol photons m^−2^ s^−1^, under 70% humidity, with 16 hours light or an 8-hour dark photoperiod.

### 2.8. RNA-Seq for Specific Tissues in Pineapple

Four stages of sepal, three stages of petal, five stages of stamen, and seven stages of ovule samples were collected from pineapple MD2 variety. Samples were stored in liquid nitrogen, and RNA was extracted through Plant RNeasy Mini Kit (Qiagen, Hilden, Germany) following manufacturer protocol. The cDNA libraries were constructed using the NEBNext Ultra™ RNA Library Prep Kit for Illumina (NEB) following standard protocols. Briefly, the mRNA was fragmented and the fragmented mRNA was converted into a double-stranded cDNA. The cDNA ends were polished and ligated to NEBNext adapters. The adaptor-ligated DNA was amplified by 15 cycles of PCR followed by purification to obtain the final library for sequencing. The DNA yield and fragment insert size distribution of the library were determined on the Agilent Bioanalyzer. The libraries were sequenced on the HiSeq 2500 sequencing instrument using 100 bp pair-end protocols at the Center for Genomics and Biotechnology in Fujian Agriculture and Forestry University [20]. RNA-Seq data from the following tissues, that is, flower, leaf, root, and six stages of fruit, was extracted from a previous study [21]. A heat expression map was developed for sixteen SBP genes to show the expression pattern in different tissues in pineapple.

Additionally, the accuracy of RNA-Seq data was confirmed through qRT-PCR. Total RNA was extracted from specific tissues (leaf, root, flower, stamen, and fruit) and qRT-PCR was run as we do for abiotic stress study (shown below).

### 2.9. Abiotic Stress Treatment

Fully grown pineapple plants (3 biological replicates) were exposed to the four different types of abiotic stresses (cold, heat, drought, and salt). With cold stress at 4°C [22] and heat stress at 45°C, 400 mMol/mL mannitol and 400 mMol/mL NaCl were applied for drought stress and salt stress, respectively, and samples were collected at 24-hour and 48-hour time points, respectively. Samples were stored in liquid nitrogen at −80°C, RNA was extracted using RNA extraction kit (Omega Bio-Tek), cDNA was extracted using cDNA extraction kit (TransGen), and qPCR was performed using qPCR kit (TransGen), while the cis-DNA-acting elements of the pineapple SBP gene promoter were carried out to test the function of SBP genes, especially against the abiotic response [23].

### 2.10. Quantitative Real-Time PCR

To investigate expression profiles of sixteen SBP genes in pineapple under abiotic stresses, pineapple (MD2) plants were exposed to four different treatments, cold, heat, drought, and mannitol for 24 h and 48 h and changes in the gene expression were analyzed using qRT-PCR. Quantitative real-time PCR was performed to determine the relative transcript levels of selected SBP family genes in pineapple according to the manufacturer's instructions for the Bio-Rad real-time PCR system (Foster City, CA, USA). PrimerQuest tool was used to design primers for SBP gene family (http://www.idtdna.com/PrimerQuest/Home/Index?Display=AdvancedParams). The actin gene (housekeeping gene) from pineapple was used as a reference. Total RNA was extracted using RNA extraction kit (Omega Bio-Tek) following manufacturer's protocol, and cDNA was synthesized from RNA by using cDNA extraction kit (TransGen) with gDNA remover. Quantitative real-time PCR (qRT-PCR) was performed using qRT-PCR kit (TransGen). Real-time PCR system in a total volume of 20 *μ*L with the following steps: denaturation at 95°C for 30 s, annealing at 95°C for 5 s, and extension at 60°C for 30 s followed by 39 cycles. SYBR green fluorescence was measured at each cycle to monitor the amplification of target SBP family genes. The Ct (threshold cycle) values were measured at each cycle, Ct (threshold cycle) is defined as the real-time PCR cycle at which a statistically significant increase in reporter fluorescence is first detected. Three biological replicates were carried out. The relative transcript levels of the analyzed SBP family genes were normalized to the transcript levels of AcoActin gene. Livak method (−ΔΔCT) was used to calculate the expression level of SBP family genes in pineapple [24].

## 3. Results

### 3.1. Identification of SQUAMOSA (SBP) Gene Family in Pineapple

To identify the SBP gene family in pineapple (*Ananas comosus*), the Hidden Markov Model (HMM) profile of the SBP domain (PF03110) was downloaded from the Pfam database. For the identification of SBP transcription factor-coding genes of *Ananas comosus*, the HMM profile of the SBP domain was used to query pineapple (*Ananas comosus*) genome database as well as nonredundant protein database with the help of the BLAST program. Sixteen SBP gene sequences were identified from the HMMER database in pineapple genome.

### 3.2. Characterization of SBP Genes in Pineapple

ExPASy server was used to calculate the isoionic point (IP) and molecular weights of SBP genes. The isoelectronic point or isoionic point (IP) is the pH, at which the amino acid does not migrate in an electric field. The lowest IP value was 6.05 for *AcSBP5*, while the highest IP value was 9.57 for *AcSBP16*. The molecular weight ranges from 22.7 to 121.9 kD for *AcSBP8* and *AcSBP2*. SBP gene, that is, *AcSBP2*, has the highest number of amino acids (1113) and ORF (3339), followed by SBP gene, that is, *AcSBP8*, having the lowest number of amino acids (210) and ORF (630) (Table 1).

### 3.3. Phylogenetic Analysis of SBP Gene Family in Pineapple

**Figure 1:**
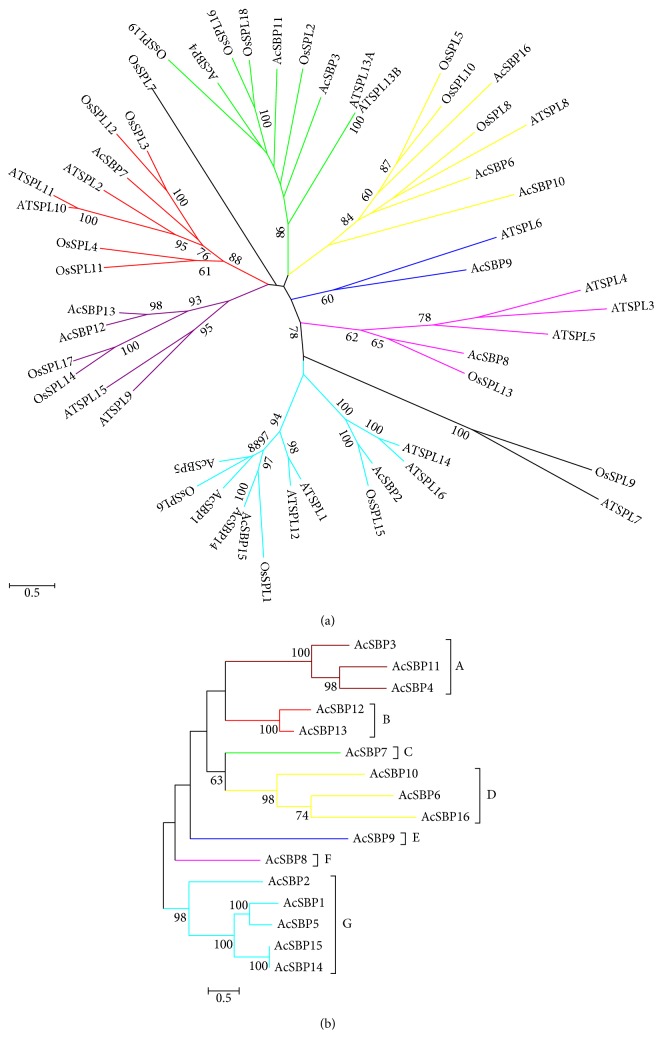
(a) Phylogenetic tree of Arabidopsis, rice, and pineapple SBP gene family. The unrooted phylogenetic tree was constructed using RAxML and maximum likelihood method. The full-length amino acid sequences were aligned by using ClustalX. The bootstrap test was performed with 1000 iterations and boot strip value of the subfamily larger than 60. AT: Arabidopsis; LOC: rice; and Aco: pineapple. (b) Phylogenetic relationship of SBP gene in pineapple genome. The full-length amino acid sequences were aligned by using MUSCLE, and a phylogenetic tree was constructed using RAxML.

Multiple sequence alignments were carried out based on full-length amino acid sequences of SBP gene family using MUSCLE software. To study the evolutionary relationship of SBP gene family in pineapple with *Arabidopsis thaliana* and rice, an unrooted phylogenetic tree was constructed using RAxML and the maximum likelihood method. The bootstrap test was performed with 1000 iterations based on the alignment of the SBP domain sequences. The phylogenetic analysis showed sixteen (16) SBP genes in pineapple (*Ananas comosus*) genome, seventeen (17) SBP genes in *Arabidopsis thaliana*, nineteen (19) SBP genes in *Oryza sativa* (Figure 1(a)). An individual phylogenetic tree was also constructed for 16 pineapple SBP gene family and divided it into 7 groups (A, B, C, D, E, F, and G) through RAxML and the maximum likelihood method [25, 26] (Figure 1(b)).

### 3.4. Calculation of Ka/Ks Values

The divergence and evolutionary relationship were calculated by Ka, Ks, and Ka/Ks ratios between SBP genes. We identified 5 paralogs in pineapple for SBP genes using bio-pipeline-master (https://github.com/tanghaibao/bio-pipeline/tree/master/synonymous_calculation). Ka/Ks ratio varied from 0.20 for *AcSBP14* and *AcSBP15* to 0.36 for *AcSBP6* and *AcSBP16* (Table 2). The trend of Ka/Ks ratio in the coding sequences of each gene pairs indicates that the SBP domains have undergone strong purifying selection (Ka/Ks < < 1) during the process of evolution. In general, Ka/Ks ratio less than 1, equal to 1, and greater than 1 means negative or stabilizing selection, neutral selection, and positive selection, respectively [27].

### 3.5. SBP Gene Location on Chromosomes

**Figure 2:**
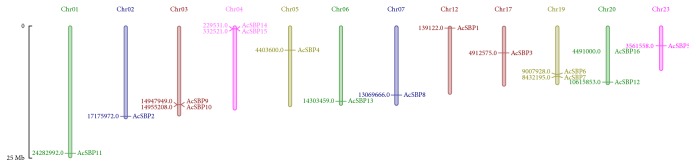
Distribution of SBP genes in pineapple genome. *Mapinspect* was used to locate genes on the chromosome. Gene start point is shown on the chromosome, while gene sizes are shown in megabases (Mb) against each gene.

To derive the SBP gene location on chromosomes, *Mapinspect* software was used to locate the SBP genes on chromosomes. It was found that sixteen SBP-box genes were located on twelve out of twenty-five chromosomes in pineapple genome: chromosomes 1, 2, 3, 4, 5, 6, 7, 12, 17, 19, 20, and 23. Two of the SBP genes were located on chromosome 20, two on chromosome 4, two on chromosome 3, and two on chromosome 19 (Figure 2).

### 3.6. Gene Structure and Conserved Motif Analysis of SBP Gene Family in Pineapple

**Figure 3:**
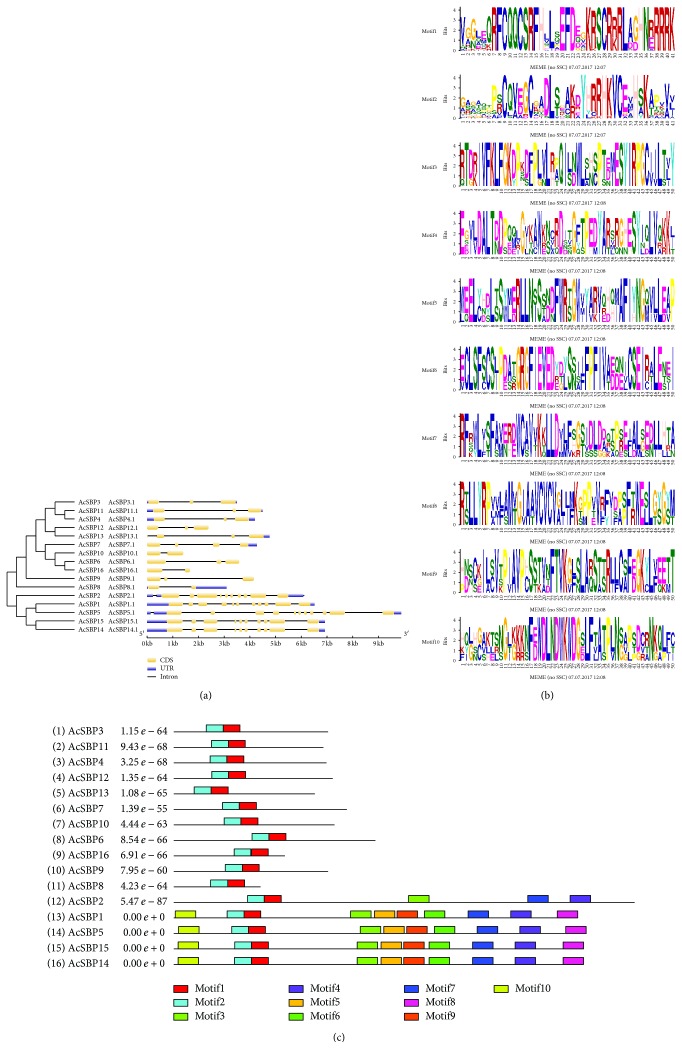
(a) Exon-intron structures of SBP genes in pineapple genome. All of the SBP genes were divided into four groups. Yellow color shows CDS (exon), blue color shows UTR (untranslated regions), and the normal line represents introns. (b) Sequence logo of SBP protein domains was obtained by MEME program. The overall height of each stack represents the degree of conservation at each position, while the height of letters within each stack indicates the relative frequency of amino acids. (c) Motif of SBP proteins in pineapple. MEME search tool was used to make motif structures.

Gene Structure Display Server was employed to analyze the structural organization of intron-exon of SBP gene family in pineapple. Gene structure analysis showed that most of the genes have the same number of introns. SBP gene family has 2 introns and 3 exons for most of the genes. Five SBP genes have the highest number of introns and exons, that is, *AcSBP2*, *AcSBP1*, *AcSBP5*, *AcSBP14*, and *AcSBP15*, indicating structural similarities (Figure 3(a)) (Table 1). Ten protein motifs were identified through MEME program (Figure 3(b)).

### 3.7. Expression Profiles of SBP Genes in Different Pineapple Tissues

To study the expression of SBP gene family in pineapple, we used these RNA-Seq data and further performed RNA-Seq analysis for four stages of sepal, three stages of petal, five stages of stamen, and seven stages of ovule [28].

**Figure 4:**
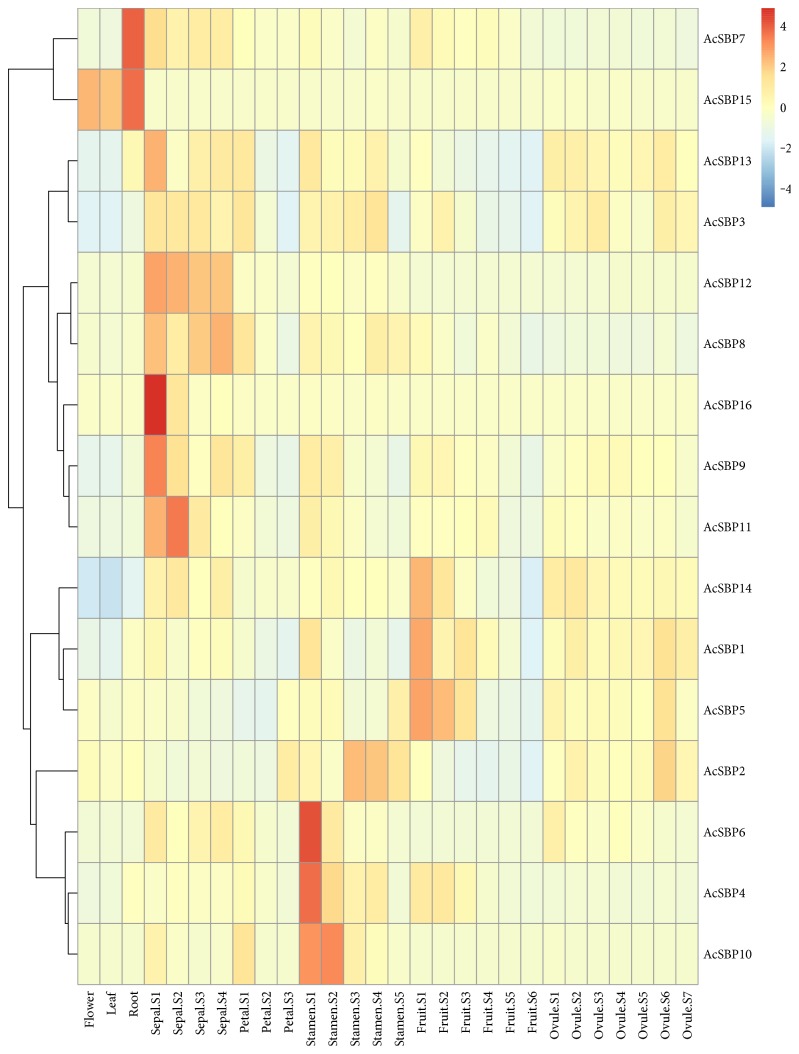
A heat map of tissue-specific expression profile of SBP genes in pineapple. Expression level can be understood using the given scale.

**Figure 5:**
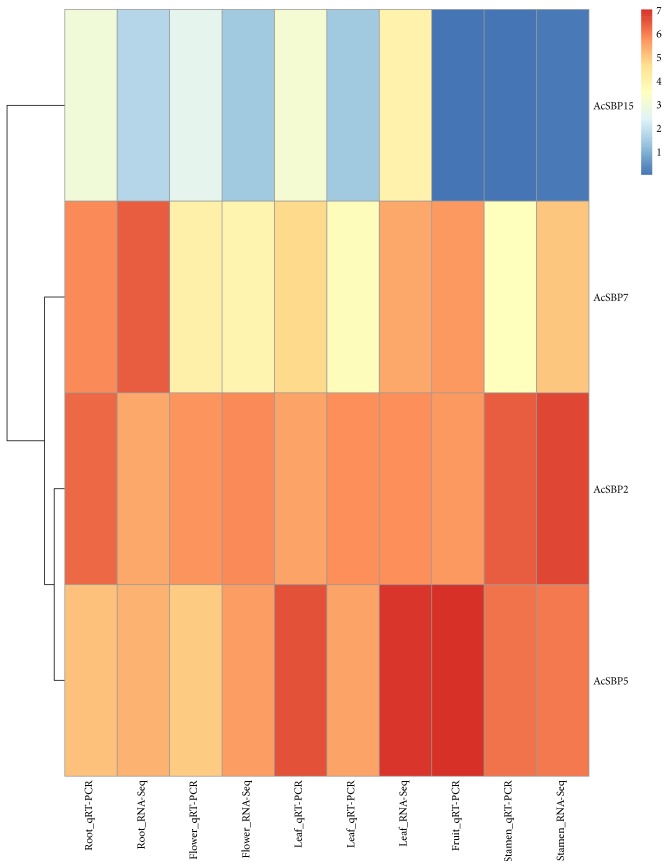
A heat map of validation of RNA-Seq of four SBP genes through qRT-PCR. The heat map was constructed using relative expression of four genes in five different tissues tested by qRT-PCR along with FPKM values of RNA-Seq of these tissues.

RNA-Seq expression profiles for SBP gene family in pineapple revealed that there was only one gene *AcSBP15* showed low expression in flower and leaf tissues, indicating SBP gene family having a little role in flower and leaf tissue development. In root tissues, two SBP genes, that is, *AcSBP7* and AcSBP15, were highly expressed. Sepal is considered as the first whorl of flower, usually green in color. It provides safety and nutrients in initial stages of flower development. At sepal developmental stage 1, two genes, that is, *AcSBP16* and *AcSBP9*, were highly expressed, while two genes, that is, *AcSBP11* and *AcSBP13*, were moderately expressed at stage 2. High expression was shown by only one gene, that is, *AcSBP11* at stage 2. On the other hand, at all four stages of sepal development, only one gene, that is, *AcSBP12*, was moderately expressed. Petals are collectively called as corolla. There was no specific SBP gene expression at the three different stages of petal development, suggesting that SBP genes may not involve in petal formation. Stamen is the male reproductive organ of flower producing pollen grains. Collectively, stamens form androecium. RNA-Seq analysis was carried out for the 5 developmental stages of stamen revealed; two genes *AcSBP4* and *AcSBP6* were highly expressed at the first developmental stage of stamen. One gene, that is, *AcSBP10*, was moderately expressed at stage 1 and stage 2. Moderate expression of SBP gene, that is, *AcSBP2*, was observed at stage 3 and 4 of stamen development. Pineapple fruit is of great economical and nutritional value as being consumed as fresh, cooked, juiced, and preserved form. RNA-Seq analysis performed for 6 stages of fruit development indicated 3 genes, that is, *AcSBP14*, *AcSBP1*, and *AcSBP5*, were moderately expressed at stage 1, followed by gene *AcSBP5* that also showed moderate expression at stage 2, while the rest of the SBP genes were not expressed at the four stages of fruit development. So, for the improvement of the pineapple crop, further studies can be done related to genes that are highly expressed in SBP genes showing high RNA-Seq expression regarding fruit development. Furthermore, ovule, plant structure that develops into a seed when fertilized. Ovule development was divided into 7 stages. RNA-Seq analysis showed no specific expression at all stages of ovule development (Figure 4()). The reads and depths obtained during RNA-Seq for each tissue are listed in additional data. The highest reads were observed for fruit developmental stage 6 (10.69 G) with the highest depths (20.32) while the lowest reads were reported for fruit developmental stage 1 (2.57 G) with sequence depths (4.88) (Supplementary Table 3s available online at https://doi.org/10.1155/2017/1032846). RNA-Seq data for four genes, that is, *AcSBP2*, *AcSBP5*, *AcSBP7*, and *AcSBP15*, were selected, and their RNA-Seq expression was confirmed through qRT-PCR in five different tissues (leaf, root, flower, stamen, and fruit). The relative expression of qRT-PCR for these genes showed consistent results with the RNA-Seq expression. The data of relative expression of these genes and FPKM data of RNA-Seq were shown using a heat map (Figure 5()).

### 3.8. Expression Pattern of SBP Genes under Cold Stress

**Figure 6:**
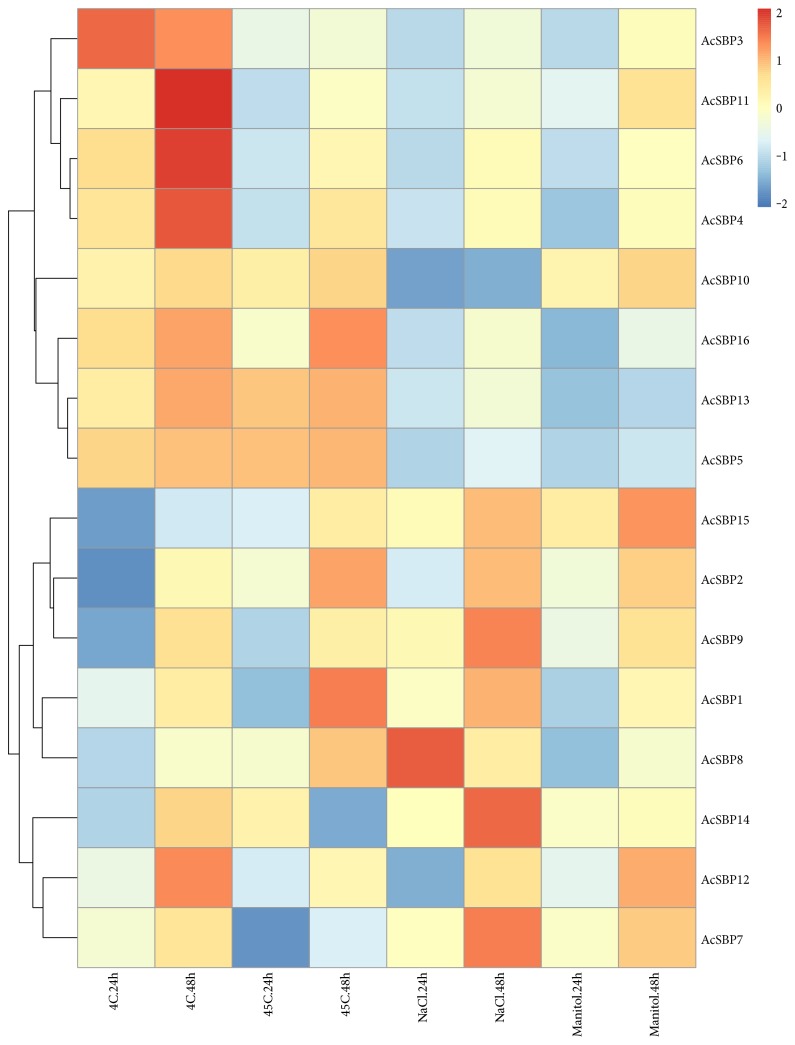
A heat map of expression profiles of SBP genes under abiotic stresses [cold (4°C), heat (45°C), salt (NaCl), and drought (mannitol)]. qRT-PCR was used to analyze the relative expression level of each SBP gene. The expression level of pineapple actin was used as the internal control to standardize RNA samples for each reaction, and the expression at 0 h was taken as 1 (data not shown). Expression level can be understood using the given scale.

Pineapple is cold sensitive and cannot withstand cold and mostly all pineapple varieties are injured after exposure to 4°C for 24 hours. After exposure to cold stress at 4°C, four genes, that is, *AcSBP11*, *AcSBP6*, *AcSBP4*, and *AcSBP12*, were highly expressed, while 3 genes, that is, *AcSBP16*, *AcSBP13*, and *AcSBP5* were moderately expressed followed by low expression of two genes, that is, *AcSBP2* and AcSBP8 after 48 h of cold treatment. On the other hand, only one gene *AcSBP3* showed high expression on 24 h as compared to 48 h treatment. The result indicates that these genes can be utilized to develop cold-resistant varieties in pineapple (Figure 6).

### 3.9. Expression Pattern of SBP Genes under Heat Stress

In this study, heat stress was applied at 45°C for 24 h and 48 h intervals, showing that after exposure to heat stress, gene expression of two genes, that is, *AcSBP16* and *AcSBP1*, were noted to be increased at 45°C after 48 h. Moreover, the rest of all SBP genes were moderately expressed after 48 h of treatment except one gene *AcSBP14*, which exhibited high expression after 24 h as compared to after 48 h. Therefore, there is no significant difference in SPB family gene expression when the plants are exposed to heat stress. That reveals that SBP family genes are not significantly responsive to heat stress (Figure 6).

### 3.10. Expression Pattern of SBP Genes under Salt Stress

To evaluate the gene expression of SBP gene family under saline conditions, pineapple plants were exposed to salt stress as 400 mMol/mL NaCl for 24 h and 48 h. Results showed that two genes, that is, *AcSBP7* and *AcSBP14*, were highly expressed, two genes, that is, *AcSBP5* and *AcSBP10*, exhibited very low gene expression on 48 h. After 48 h of treatment, the expression pattern of four genes, that is, *AcSBP2*, *AcSBP9*, *AcSBP1*, and *AcSBP15*, was moderate while only one gene *AcSBP8* was highly expressed on 24 h but reduced expression was observed on 48 h. This dynamic response of SBP genes to salt stress predicts an important role of SBP genes in response to salt stress (Figure 6).

### 3.11. Expression Pattern of SBP Genes under Drought Stress

Pineapple plants were subjected to drought stress (mannitol in concentration of 400 mMol/mL). For drought stress, two genes, that is, *AcSBP12* and *AcSBP15*, exhibited consistent high gene expression as compared to other SBP genes which were moderately expressed followed by very low expression of one gene *AcSBP16* after 48 h of treatment. On the basis of these findings, an innovative role of SBP genes can be predicted against drought stress (Figure 6). The cis-DNA-acting elements of the pineapple SBP gene promoter were carried out to test the function of SBP genes, especially the abiotic response [23] (supplementary Table 1s).

## 4. Discussion

Pineapple is the most economically important plant in the Bromeliaceae family [1, 2]. SQUAMOSA promoter-binding protein (SBP-box) gene family encodes SBP proteins which play a vital role in plant development, signaling, and defense mechanisms. Therefore, it is important to study the characterization, analysis, and gene expression profiles of SBP gene family through genome-wide data in response to abiotic stresses in pineapple. This study was focused on 16 SBP genes in pineapple genome.

### 4.1. Phylogenetic Analysis, Gene Structure, Identification of Conserved Protein Motifs, and Localization of Chromosomes

SBP gene family in pineapple was shown along with *Arabidopsis thaliana* and rice, showing high similarity and conservation (Figure 1(a)). SBP gene family could be divided into seven groups on the basis of structural similarities (Figure 1(b)). SBP gene family could be divided into six groups in pepper on the basis of phylogenetic analysis [29]. SBP gene family was divided into four groups in melon [30]. The structural organization of intron-exon showed high variation ranged from 1 to 11 in one gene, similar as in Arabidopsis and rice genome (Table 1). Maximum number of introns reported in pepper ranged from 0 to 11 [29] and in melon, ranged from 0 to 10 [30].

To further highlight the structure of SBP gene family, this study showed ten protein motifs, suggesting that SBP gene family was highly conserved in pineapple as the same as in rice and Arabidopsis (Figure 3(c)). High conservation among protein motifs in SBP gene family was also observed in melon, pepper, and apple [4, 29, 30]. The resemblance and structural variation among these motifs can be further studied to provide novel information. The results showed that SBP genes were located on 12 chromosomes in pineapple genome while chromosome 4, 3, 19, and 20 contains more than one SBP genes (Figure 2). Ka, Ks, and Ka/Ks ratios between SBP genes showed divergence and evolutionary relationship among paralogs (Table 2). In general, Ka/Ks ratio less than 1, equal to 1, and greater than 1 means negative or stabilizing selection, neutral selection, and positive selection, respectively.

### 4.2. RNA-Seq Analysis in Different Tissues

RNA-Seq is used as a powerful tool to study gene expression profiles. In this study, RNA-Seq was carried out to analyze the expression pattern of sixteen SBP genes in pineapple. We used RNA-Seq data of flower, leaf, root, and six stages of fruit provided by [21] and performed RNA-Seq analysis for further tissues, that is, four stages of sepal, three stages of petal, five stages of stamen, and seven stages of ovule (Figure 4). RNA-Seq analysis revealed that only one SBP gene showed low expression in flower and leaf tissues. High expression was shown by two SBP genes in root, indicating a vital role of SBP genes in the development of root. At developmental stages of sepal S1 and S2, high and moderate expressions of SBP genes were observed. No specific expression was noted at the three stages of development in petal, showing no role of SBP gene family in petal formation. Two SBP genes *AcSBP4* and *AcSBP6* were highly expressed at stamen S1 stage followed by moderate expression of only one SBP gene at stamen S1 and S2 stages while at stamen S3 and S4 stage, only one gene was moderately expressed. These SBP genes can be used to improve flower reproductive development in pineapple. Four SBP genes, that is, *AcSBP14*, *AcSBP1*, *AcSBP5*, and *AcSBP5*, showed high expression at the S1 and S2 stages of fruit development while none of the genes were expressed at other four stages of fruit development. So, these SBP genes can be used to improve the pineapple varieties to have more yields because pineapple fruit is of great importance containing bromelain, a digestive enzyme. RNA-Seq analysis showed that SBP genes were not specifically expressed in the developmental stages of ovule. Therefore, it is possible to suggest that SBP gene family have no specific role in the development of ovule (Figure 4). The above finding was supported up to some extent in pepper where there one SBP gene *AcSBP02* had expression in root, leaf, green fruit, and mature fruit but no expression was observed in flower in pepper [29]. Our RNA-Seq was accurate as we confirmed it through qRT-PCR. RNA-Seq expression of different genes in different tissues was confirmed through qRT-PCR; the results were consistent with the RNA-Seq data.

### 4.3. Response of SBP Genes to Abiotic Stress

Recent studies suggested that SBP-box genes are involved in defense mechanism and signal transduction, showing response to biotic and abiotic stress. AtSPL14 has been involved in programmed cell death, inducing fungal toxin fumonisin B1 [15]. *AtSPL* genes are coexpressed with two TFs, TGA1, and WRKY65 which can be induced by pathogens and regulate the expression of genes which are considered to be involved in response to stress, such as pathogenesis-related 1 (PR-1) protein and glutathione s-transferase 6 (GST6) [31].

In response to cold stress treatment, only one gene was highly expressed at 24 h while four genes were highly expressed at 48 h of treatment. Pineapple is sensitive to cold, so these genes can be further used in development of cold-stress resistance varieties. Pineapple is a tropical fruit crop that can grow in high temperatures. The results showed no specific expression to heat stress with some exceptions; only one gene was highly upregulated at 24 h while two genes were highly upregulated at 48 h treatment. These results indicate that SBP genes show resistance to high temperature in pineapple because it is a tropical and subtropical plant where the temperature ranges from 25 to 40°C in summer. Salinity is considered one of the major environmental stresses that adversely affect productivity of agricultural crops. Saline conditions cause decrease in crop yield, length of shoot, root, stem, and leaves. After exposure to salt stress, one gene *AcSBP8* showed high expression at 24 h while two genes, that is, *AcSBP7* and *AcSBP14*, were noted to be highly expressed at 48 h of treatment and magnitude of expression was too high, indicating that these two genes having a key role in response to salt stress, so it is suggested that these genes can be used in pineapple variety development against salt stress and can also be used in gene expression studies to evaluate its function. Drought is the most important water-related problem caused by annual precipitation, declining ground water levels at faster rate, and insufficient water for plant growth and development. After exposure to drought stress, only two genes, that is, *AcSBP12* and *AcSBP15*, were highly upregulated at 48 h and rate of expression was higher, showing the vital role of these genes to drought stress focusing that these genes can be used in transformation processes to develop drought-resistant varieties for the development of industry which is beneficial for the farming community. While the cis-DNA-acting elements of the pineapple SBP gene promoter were carried out to test the function of SBP genes, especially against the abiotic response, 16 cis-DNA acting elements were identified; these elements are playing a vital role in different biological and physiological phenomenon during plant growth (supplementary Table 2s).

SBP gene family was previously studied in pepper, melon, apple, and other species against biotic stresses, and it investigated that SBP gene family show response to biotic stresses. The purpose of this study was to investigate the response of SBP gene family against abiotic stress (cold, heat, salt, and drought). This study is the novel determination of SBP gene family against abiotic stresses coupled with RNA sequencing in pineapple. These findings suggest that SBP gene family is involved in response to abiotic stresses. Dynamic response of SBP gene family to abiotic stress has showed its vital role in various physiological and stress stimuli.

## 5. Conclusions

A comprehensive analysis of SBP gene family was carried out in pineapple. A total of sixteen SBP genes were identified in pineapple genome. Furthermore, these 16 SBP genes were classified according to phylogenetic tree, gene structure, conserved amino acid sequences, protein motifs, high-throughput sequencing, and RNA-Seq expression profiles for specific tissue at different developmental stages in pineapple genome. RNA-Seq was also validated through qRT-PCR. SBP genes were characterized by different traits, that is, molecular weight, isoelectric point, ORF, number of intron-exon, and number of amino acids. Also, this study accompanied by the effect of SBP genes against various abiotic stresses (cold, heat, salt, and drought) and expression profiles were analyzed by qRT-PCR. *cis*-DNA-acting elements were also identified. The results obtained from this investigation are useful information to highlight the molecular background of SBP gene family in pineapple. The results obtained were according to our expectations and the information published related to SBP gene family studies in other species but we consider that SBP gene family response to abiotic stress is a different parameter that gives a unique understanding of SBP gene family and their function in different tissues. Our study has the efficacy for understanding how SBP genes can be utilized for its gene function and pineapple crop improvement.

## Supplementary Material

Supplementary Table. 1s The cis-DNA acting elements of the pineapple SBP genes. Supplementary Table. 2s Functions of cis-DNA acting elements of the pineapple SBP genes. Supplementary Table. 3s Reads obtained from RNA-Seq analysis (sequencing depth).

## Figures and Tables

**Table 1:** Characterization of SQUAMOSA promoter-binding protein (SBP) gene family in pineapple.

Gene name	Gene ID	IP	MV (kDa)	Amino acid	ORF	Chr.	Introns	Exons
*Aco000696.1*	*AcSBP1*	6.5	108.1	975	2925	12	10	11
*Aco000726.1*	*AcSBP2*	7.9	121.9	1113	3339	2	10	10
*Aco003668.1*	*AcSBP3*	6.7	39.7	373	1119	17	2	3
*Aco004608.1*	*AcSBP4*	8.7	40.4	369	1107	5	2	3
*Aco007331.1*	*AcSBP5*	6.1	109.5	996	2988	23	11	11
*Aco008265.1*	*AcSBP6*	6.6	52.4	487	1461	19	2	3
*Aco008321.1*	*AcSBP7*	9.3	44.7	418	1254	19	3	4
*Aco010539.1*	*AcSBP8*	9.4	22.7	210	630	7	1	2
*Aco012822.1*	*AcSBP9*	9	40.2	372	1116	3	2	3
*Aco012823.1*	*AcSBP10*	7.6	41.3	388	1164	3	1	2
*Aco015107.1*	*AcSBP11*	8.6	39.7	362	1086	1	2	3
*Aco015338.1*	*AcSBP12*	9.1	39.4	384	1152	20	2	3
*Aco018505.1*	*AcSBP13*	8.9	36.5	341	1023	6	3	3
*Aco021992.1*	*AcSBP14*	6.1	110.7	989	2967	4	9	10
*Aco023516.1*	*AcSBP15*	6.2	110.8	989	2967	4	9	10
*Aco031754.1*	*AcSBP16*	9.6	30.2	269	807	20	1	2

IP: isoelectric point; MV: molecular weight; ORF: open reading frame; Chr.: chromosome number.

**Table 2:** Ka/Ks ratios of SBP genes.

Gene ID	Gene ID	Ks	Ka	Ka/Ks ratio	Selection
*AcSBP1*	*AcSBP5*	1.1931	0.2934	0.245914	Purifying
*AcSBP4*	*AcSBP11*	1.6692	0.4106	0.245986	Purifying
*AcSBP6*	*AcSBP16*	1.5905	0.5773	0.362968	Purifying
*AcSBP12*	*AcSBP13*	1.2578	0.2592	0.206074	Purifying
*AcSBP14*	*AcSBP15*	0.0045	0.0009	0.20	Purifying

## Abbreviations

TFs: Transcription factors
RNA-Seq: RNA sequencing
SBP: Squamosa binding protein
NaCl: Sodium chloride
Aco: Pineapple.

## References

[1] Firoozabady E., Gutterson N. (2003). Cost-effective in vitro propagation methods for pineapple. *Plant Cell Reports*.

[2] Gangopadhyay S., Harding B. L., Rajagopalan B., Lukas J. J., Fulp T. J. (2009). A nonparametric approach for paleohydrologic reconstruction of annual streamflow ensembles. *Water Resources Research*.

[3] Escalona M., Lorenzo J. C., González B. (1999). Pineapple (*Ananas comosus* L. Merr) micropropagation in temporary immersion systems. *Plant Cell Reports*.

[4] Li J., Hou H., Li X. (2013). Genome-wide identification and analysis of the SBP-box family genes in apple (Malus × domestica Borkh.). *Plant Physiology and Biochemistry*.

[5] Klein J., Saedler H., Huijser P. (1996). A new family of DNA binding proteins includes putative transcriptional regulators of the Antirrhinum majus floral meristem identity gene SQUAMOSA. *Molecular & General Genetics*.

[6] Cardon G., Höhmann S., Klein J., Nettesheim K., Saedler H., Huijser P. (1999). Molecular characterisation of the *Arabidopsis* SBP-box genes. *Gene*.

[7] Cardon G. H., Hohmann S., Nettesheim K., Saedler H., Huijser P. (1997). Functional analysis of the *Arabidopsis thaliana* SBP-box gene *SPL3*: a novel gene involved in the floral transition. *The Plant Journal*.

[8] Lannenpaa M., Janonen I., Holtta-Vuori M., Gardemeister M., Porali I., Sopanen T. (2004). A new SBP-box gene *BpSPL1* in silver birch (*Betula pendula*). *Physiologia Plantarum*.

[9] Zhang B., Liu X., Zhao G., Mao X., Li A., Jing R. (2014). Molecular characterization and expression analysis of *Triticum aestivum* squamosa-promoter binding protein-box genes involved in ear development. *Journal of Integrative Plant Biology*.

[10] Xie K., Wu C., Xiong L. (2006). Genomic organization, differential expression, and interaction of SQUAMOSA promoter-binding-like transcription factors and microRNA156 in rice. *Plant Physiology*.

[11] Chuck G. S., Tobias C., Sun L. (2011). Overexpression of the maize Corngrass1 microRNA prevents flowering, improves digestibility, and increases starch content of switchgrass. *Proceedings of the National Academy of Sciences of the United States of America*.

[12] Salinas M., Xing S., Höhmann S., Berndtgen R., Huijser P. (2012). Genomic organization, phylogenetic comparison and differential expression of the SBP-box family of transcription factors in tomato. *Planta*.

[13] Hou H., Li J., Gao M. (2013). Genomic organization, phylogenetic comparison and differential expression of the SBP-box family genes in grape. *PLoS One*.

[14] Zhang X., Dou L., Pang C. (2015). Genomic organization, differential expression, and functional analysis of the SPL gene family in Gossypium hirsutum. *Molecular Genetics and Genomics*.

[15] Stone J. M., Liang X., Nekl E. R., Stiers J. J. (2005). Arabidopsis *AtSPL14*, a plant-specific SBP-domain transcription factor, participates in plant development and sensitivity to fumonisin B1. *The Plant Journal*.

[16] Mittler R. (2006). Abiotic stress, the field environment and stress combination. *Trends in Plant Science*.

[17] Kawahara Y., de la Bastide M., Hamilton J. P. (2013). Improvement of the *Oryza sativa* Nipponbare reference genome using next generation sequence and optical map data. *Rice*.

[18] Tamura K., Peterson D., Peterson N., Stecher G., Nei M., Kumar S. (2011). MEGA5: molecular evolutionary genetics analysis using maximum likelihood, evolutionary distance, and maximum parsimony methods. *Molecular Biology and Evolution*.

[19] Guo A.-Y., Zhu Q.-H., Chen X., Luo J.-C. (2007). GSDS: a gene structure display server. *Yi chuan = Hereditas*.

[20] Cai H., Zhao L., Wang L. (2017). ERECTA signaling controls *Arabidopsis* inflorescence architecture through chromatin-mediated activation of *PRE1* expression. *The New Phytologist*.

[21] Ming R., VanBuren R., Wai C. M. (2015). The pineapple genome and the evolution of CAM photosynthesis. *Nature Genetics*.

[22] Chen C., Zhang Y., Xu Z. (2016). Transcriptome profiling of the pineapple under low temperature to facilitate its breeding for cold tolerance. *PLoS One*.

[23] Lescot M., Déhais P., Thijs G. (2002). PlantCARE, a database of plant cis-acting regulatory elements and a portal to tools for in silico analysis of promoter sequences. *Nucleic Acids Research*.

[24] Livak K. J., Schmittgen T. D. (2001). Analysis of relative gene expression data using real-time quantitative PCR and the 2^−ΔΔC^_T_ method. *Methods*.

[25] Kushwaha H., Gupta S., Singh V. K., Rastogi S., Yadav D. (2011). Genome wide identification of *Dof* transcription factor gene family in sorghum and its comparative phylogenetic analysis with rice and Arabidopsis. *Molecular Biology Reports*.

[26] Lijavetzky D., Carbonero P., Vicente-Carbajosa J. (2003). Genome-wide comparative phylogenetic analysis of the rice and Arabidopsis Dof gene families. *BMC Evolutionary Biology*.

[27] Cannon S. B., Mitra A., Baumgarten A., Young N. D., May G. (2004). The roles of segmental and tandem gene duplication in the evolution of large gene families in *Arabidopsis thaliana*. *BMC Plant Biology*.

[28] Su Z., Wang L., Li W. (2017). Genome-wide identification of auxin response factor (ARF) genes family and its tissue-specific prominent expression in pineapple (*Ananas comosus*). *Tropical Plant Biology*.

[29] Zhang H.-X., Jin J.-H., He Y.-M. (2016). Genome-wide identification and analysis of the SBP-box family genes under *Phytophthora capsici* stress in pepper (Capsicum annuum L.). *Frontiers in Plant Science*.

[30] Ma Y., Guo J. W., Bade R., Men Z. H., Hasi A. (2014). Genome-wide identification and phylogenetic analysis of the SBP-box gene family in melons. *Genetics and Molecular Research*.

[31] Wang Y., Hu Z., Yang Y., Chen X., Chen G. (2009). Function annotation of an SBP-box gene in *Arabidopsis* based on analysis of co-expression networks and promoters. *International Journal of Molecular Sciences*.

