# Supplementary material for: Genomic Survey, Characterization, and Expression Profile Analysis of the SBP Genes in Pineapple (Ananas comosus L.)
Source: Int J Genomics. 2017 Sep 29;2017:1032846. doi: 10.1155/2017/1032846 (PMC5643045; doi:10.1155/2017/1032846)
Supplement: Supplementary file 1 — Supplementary Table. 1s The cis-DNA acting elements of the pineapple SBP genes. Supplementary Table. 2s Functions of cis-DNA acting elements of the pineapple SBP genes. Supplementary Table. 3s Reads obtained from RNA-Seq analysis (sequencing depth). [file 1032846.f1.doc]

Supplementary Table. 1s

The cis-DNA acting elements of the pineapple SBP genes.

| Gene name | ID | AuxRR-core | CGTCA-motif | CCAAT | EIRE | ERE | GARE-motif | GCC-box | HSE | LTR | MBS | TC-rich | TCA-element | TGA-element | W -box | WUN-motif |
| --- | --- | --- | --- | --- | --- | --- | --- | --- | --- | --- | --- | --- | --- | --- | --- | --- |
| *Aco000696.1* | *AcSBP1* | 1 |  |  |  |  |  |  |  | 3 | 2 | 2 | 2 | 1 | 1 | 1 |
| *Aco000726.1* | *AcSBP2* |  |  |  | 1 |  |  |  | 1 |  | 1 | 1 | 3 | 1 | 1 |  |
| *Aco003668.1* | *AcSBP3* |  |  |  |  |  | 1 |  | 1 |  |  |  | 1 |  |  |  |
| *Aco004608.1* | *AcSBP4* |  |  | 2 | 1 | 1 | 1 |  | 1 |  |  |  | 3 |  | 1 |  |
| *Aco007331.1* | *AcSBP5* |  |  |  |  |  | 2 |  | 1 | 2 | 2 |  |  |  |  |  |
| *Aco008265.1* | *AcSBP6* |  |  |  |  |  |  |  |  |  |  | 1 | 5 |  |  |  |
| *Aco008321.1* | *AcSBP7* |  | 1 |  | 1 |  |  |  | 1 | 2 | 1 |  | 1 |  | 1 |  |
| *Aco010539.1* | *AcSBP8* |  |  |  | 1 | 2 |  |  | 5 |  |  | 2 | 1 |  | 1 |  |
| *Aco012822.1* | *AcSBP9* |  |  |  | 1 | 2 |  |  | 5 |  |  | 2 | 1 |  | 1 |  |
| *Aco012823.1* | *AcSBP10* |  | 1 |  |  | 1 |  |  | 1 |  | 1 |  | 1 |  |  |  |
| *Aco015107.1* | *AcSBP11* |  |  |  |  |  | 1 |  | 2 |  | 2 | 1 | 2 |  |  |  |
| *Aco015338.1* | *AcSBP12* |  | 2 |  |  |  | 1 |  | 2 | 1 |  | 1 |  |  |  |  |
| *Aco018505.1* | *AcSBP13* |  | 2 |  |  | 1 |  |  | 1 |  |  |  | 2 |  |  |  |
| *Aco021992.1* | *AcSBP14* |  |  |  |  |  | 1 |  |  | 2 | 1 |  | 3 | 1 |  |  |
| *Aco023516.1* | *AcSBP15* |  |  |  |  |  | 1 |  |  | 2 | 1 | 1 | 3 | 1 |  |  |
| *Aco031754.1* | *AcSBP16* |  | 1 |  |  |  | 1 |  | 2 |  | 1 | 1 | 3 | 1 |  |  |

Supplementary Table. 2s

Functions of cis-DNA acting elements of the pineapple SBP genes.

| S.NO | Cis-regulatory elements | Functions/description |
| --- | --- | --- |
| 1 | ABRE | cis-acting element involved in the abscisic acid responsiveness |
| 2 | AuxRR-core | cis-acting regulatory element involved in auxin responsiveness |
| 3 | CGTCA-motif | cis-acting regulatory element involved in MeJA-responsiveness |
| 4 | CCAAT | MYBHv1 binding site |
| 5 | EIRE | elicitor-responsive element |
| 6 | ERE | ethylene-responsive element |
| 7 | GARE-motif | gibberellin-responsive element |
| 8 | GCC-box | ethylene-responsive element |
| 9 | HSE | cis-acting element involved in heat stress responsiveness |
| 10 | LTR | cis-acting element involved in low-temperature responsiveness |
| 11 | MBS | MYB binding site involved in drought-inducibility |
| 12 | TC-rich | cis-acting element involved in defense and stress responsiveness |
| 13 | TCA-element | cis-acting element involved in salicylic acid responsiveness |
| 14 | TGA-element | Auxin-responsive element |
| 15 | W -box | cis-regulatory element sequence involved in deoxyribonucleic acid (DNA) |
| 16 | WUN-motif | wound-responsive element |

Supplementary Table. 3s

Reads obtained from RNA-Seq analysis (sequencing depth).

| Sample | Total Bases (G) | Sequencing Depth | Sample | Total Bases (G) | Sequencing Depth |
| --- | --- | --- | --- | --- | --- |
| Root | 6.62 | 12.58555133 | Stamen.S1 | 3.4 | 6.463878327 |
| Flower | 7.42 | 14.10646388 | Stamen.S2 | 4.6 | 8.745247148 |
| Leaf | 4.15 | 7.88973384 | Stamen.S3 | 3.9 | 7.414448669 |
| Ovule.S1 | 5 | 9.505703422 | Stamen.S4 | 4 | 7.604562738 |
| Ovule.S2 | 5 | 9.505703422 | Stamen.S5 | 4.8 | 9.125475285 |
| Ovule.S3 | 5 | 9.505703422 | Petal.S1 | 4.6 | 8.745247148 |
| Ovule.S4 | 4.9 | 9.315589354 | Petal.S2 | 4 | 7.604562738 |
| Ovule.S5 | 5.2 | 9.885931559 | Petal.S3 | 4.3 | 8.174904943 |
| Ovule.S6 | 5.1 | 9.69581749 | Fruit.S1 | 2.57 | 4.885931559 |
| Ovule.S7 | 5.3 | 10.07604563 | Fruit.S2 | 5.57 | 10.58935361 |
| Sepal.S1 | 4.7 | 8.935361217 | Fruit.S3 | 5.58 | 10.60836502 |
| Sepal.S2 | 4.5 | 8.55513308 | Fruit.S4 | 6.85 | 13.02281369 |
| Sepal.S3 | 5.2 | 9.885931559 | Fruit.S5 | 4.97 | 9.448669202 |
| Sepal.S4 | 5.3 | 10.07604563 | Fruit.S6 | 10.69 | 20.32319392 |
